# Treatment of neonatal jaundice with filtered sunlight in Nigerian neonates: study protocol of a non-inferiority, randomized controlled trial

**DOI:** 10.1186/1745-6215-14-446

**Published:** 2013-12-28

**Authors:** Tina M Slusher, Bolajoko O Olusanya, Hendrik J Vreman, Ronald J Wong, Ann M Brearley, Yvonne E Vaucher, David K Stevenson

**Affiliations:** 1 Center for Global Health, Department of Pediatrics, University of Minnesota, Minneapolis, MN, USA; 2 Department of Pediatrics, Hennepin County Medical Center, Minneapolis, MN, USA; 3 Center for Healthy Start Initiative, Dolphin Estate, Ikoyi, Lagos, Nigeria; 4 Neonatal and Developmental Medicine Laboratory, Division of Neonatology, Department of Pediatrics, Stanford University Medical Center, Stanford, CA, USA; 5 Biostatistical Design and Analysis Center, Clinical and Translational Science Institute, University of Minnesota Academic Health Center, Minneapolis, MN, USA; 6 Division of Neonatal/Perinatal Medicine, School of Medicine, University of California at San Diego, San Diego, CA, USA

**Keywords:** Filtered sunlight phototherapy, Hyperbilirubinemia, Developing country, Low-cost technologies, Irradiance, Africa

## Abstract

**Background:**

Severe neonatal jaundice and its progression to kernicterus is a leading cause of death and disability among newborns in poorly-resourced countries, particularly in sub-Saharan Africa. The standard treatment for jaundice using conventional phototherapy (CPT) with electric artificial blue light sources is often hampered by the lack of (functional) CPT devices due either to financial constraints or erratic electrical power. In an attempt to make phototherapy (PT) more readily available for the treatment of pathologic jaundice in underserved tropical regions, we set out to test the hypothesis that filtered sunlight phototherapy (FS-PT), in which potentially harmful ultraviolet and infrared rays are appropriately screened, will be as efficacious as CPT.

**Methods/design:**

This prospective, non-blinded randomized controlled non-inferiority trial seeks to enroll infants with elevated total serum/plasma bilirubin (TSB, defined as 3 mg/dl below the level recommended by the American Academy of Pediatrics for high-risk infants requiring PT) who will be randomly and equally assigned to receive FS-PT or CPT for a total of 616 days at an inner-city maternity hospital in Lagos, Nigeria. Two FS-PT canopies with pre-tested films will be used. One canopy with a film that transmits roughly 33% blue light (wavelength range: 400 to 520 nm) will be used during sunny periods of a day. Another canopy with a film that transmits about 79% blue light will be used during overcast periods of the day. The infants will be moved from one canopy to the other as needed during the day with the goal of keeping the blue light irradiance level above 8 μW/cm^2^/nm.

Primary outcome*:* FS-PT will be as efficacious as CPT in reducing the rate of rise in bilirubin levels. Secondary outcome: The number of infants requiring exchange transfusion under FS-PT will not be more than those under CPT.

**Conclusion:**

This novel study offers the prospect of an effective treatment for infants at risk of severe neonatal jaundice and avoidable exchange transfusion in poorly-resourced settings without access to (reliable) CPT in the tropics.

**Trial registration:**

ClinicalTrials.gov Identifier: NCT01434810

## Background

Severe neonatal jaundice (NNJ) or hyperbilirubinemia and its progression to acute bilirubin encephalopathy (ABE) and kernicterus is a leading, yet preventable, cause of newborn re-hospitalizations, deaths, and disabilities globally [1-5]. Phototherapy (PT), which involves exposing a newborn’s skin to electric lamp-generated blue light, is the standard treatment for removing excessive bilirubin, except in extreme cases when exchange transfusion becomes necessary [6].

Numerous studies from poorly-resourced countries suggest that severe NNJ represents perhaps the largest unrecognized cause of neonatal morbidity and mortality in the world [4,7]. In sub-Saharan Africa, especially in Nigeria and Kenya, NNJ is a leading cause of death in newborn nurseries [8-13] and long-term neurological impairment in survivors [14-18]. Unfortunately, PT may not be available in these countries because of the lack of devices and/or of reliable electrical power [19,20]. In these areas, modern PT devices are not readily affordable, often break down because of electrical power surges, and are difficult to maintain due to the unavailability of replacement parts. Even where PT devices are available, most hospitals lack the resources necessary to replace fluorescent lamps, which is recommended after 2,000 to 3,000 hours of use, and, as a consequence, simply leave ineffective tubes in place until they burn out. Moreover, very few hospitals have appropriate irradiance meters for measuring the intensity of the blue light emitted by the lamps, resulting in few or no devices providing the optimal level of irradiance required for intensive (>30 μW/cm^2^/nm) conventional phototherapy (CPT) [21,22]. However, it is not uncommon, especially in areas without access to CPT, for the parents/guardians of jaundiced infants to place their babies in direct sunlight unaware of the potential harm or safety risks.

The scientific potential of sunlight as a possible treatment for NNJ was first demonstrated by Cremer and colleagues in 1958 [23]. His team found that placing naked infants in sunlight decreased bilirubin levels. This observation led to the production of the first PT device using fluorescent blue light tubes. These early studies subsequently led to the development of commercially available, easily controlled, electricity-requiring artificial blue light sources for effective, on-demand PT of newborns in industrialized countries. The potential efficacy of direct sunlight PT compared with CPT has also been demonstrated in an *in vitro* study [24].

Using direct sunlight for PT has a number of clinical and practical drawbacks that could make its use undesirable. Sunlight contains altitude-, seasonal-, and time-of-day-dependent levels of harmful ultraviolet A, B, and C radiation, which can seriously and permanently damage human skin. It also contains significant levels of warming infrared radiation, which, in the absence of sufficient cooling, could raise core body temperatures to unsafe levels. However, several technological solutions exist for filtering unwanted radiation from any light source, including sunlight, while preserving the desirable attributes of a given energy spectrum [25]. When filtered to exclude the harmful spectral radiation, the use of sunlight can be valuable in environments that have no access to electric lamp PT.

**Figure 1:**
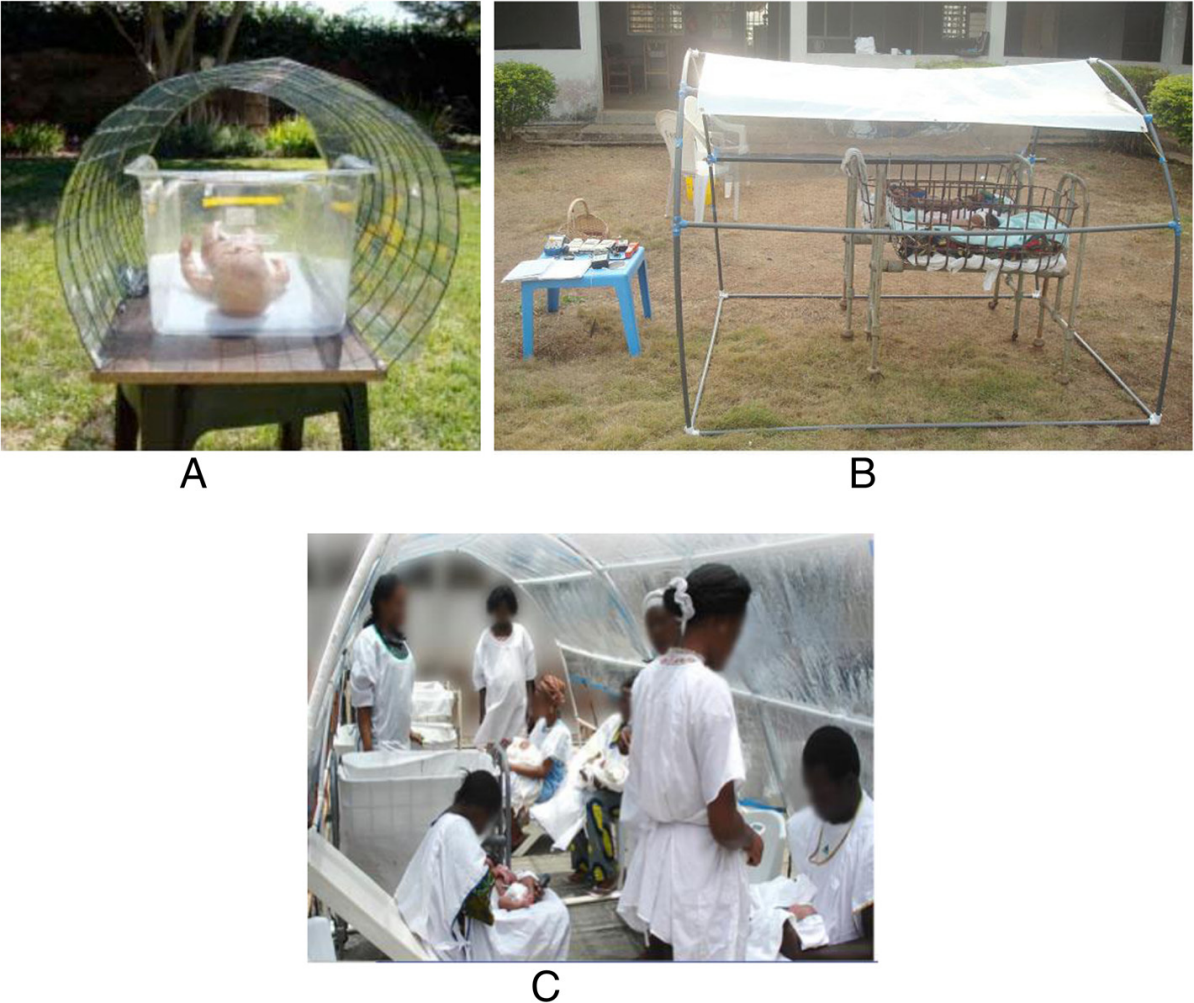
**Filtered sunlight canopies. A**: Experimental filtered sunlight canopy with a baby doll; **B**: Baby placed under a filtered sunlight canopy in an open lawn in a primary care setting; **C**: Mother-infant pairs with health workers under a group filtered sunlight canopy in a hospital setting.

The most practical filters of sunlight are the commercially available window-tinting films, widely used in vehicles and residential and commercial structures in sunny climates. Although window-tinting films are traditionally affixed to a glass surface, these films can also be stretched over a support frame, under which an infant basket (Figure 1A), bassinet, or crib (Figure 1B) can be placed. Our preliminary laboratory bench studies in California and field studies in Nigeria have shown that such films effectively remove potentially harmful radiation, while allowing the transmission of beneficial blue light required for effective PT. The levels of irradiance recorded exceeded that delivered by the most potent newborn PT devices. In one of the field tests in a rural hospital in Nigeria, seven jaundiced infants were placed under portable individual or group filtered sunlight PT (FS-PT) using film-covered canopies placed in direct sunlight in the hospital courtyard (Figure 1B,C). Body temperature and blue light irradiance were monitored every hour, and the infants were watched closely for the development of clinical dehydration and sunburn. FS-PT was tolerated well by both newborns and their mothers and allowed for maternal bonding during treatment. None of the infants developed significant hypothermia (defined as <35.5°C), and displayed no evidence of dehydration or sunburn. Six of the infants had at least one temperature episode >38.0°C during their course of FS-PT, but none exceeded 39°C, and all recovered after being returned indoors. The average time to being able to return to FS-PT was 19.7 minutes, with only two instances over 60 minutes. Moreover, placing infants on a moistened towel during high ambient temperatures (>40°C) quite readily maintained body temperatures of infants in cribs.

### Observational study on the safety and potential efficacy of filtered sunlight phototherapy

A recent comprehensive systematic review of available evidence worldwide on PT found no randomized controlled trials (RCTs) dealing with either sunlight or environmental light [26]. Prior to our proposed RCT to establish the effectiveness of FS-PT compared with CPT in a larger sample of infants, we conducted an observational study to evaluate safety and potential efficacy of two previously tested films for use in an inner-city maternity hospital in Lagos, Nigeria. FS-PT safety was determined through close monitoring of infant temperature, hydration status, and skin for signs of possible sunburn. Therapy was deemed safe on a given day if the infant did not have to be removed from phototherapy due to needing physician treatment for sunburn or dehydration, due to persistent temperature instability defined as two or more episodes of temperatures <35.0 or >39.0°C, or due to failure to return to normothermia (defined as 35.5 to 38.0°C) within 1 hour of being removed from FS-PT. Efficacy was evaluated by measuring the rate of rise/decline in total serum/plasma bilirubin (TSB) levels. Treatment was deemed safe and efficacious if the infant was able to stay in the FS-PT canopy for ≥5 hours per day and the rate of rise of TSB was <0.2 mg/dl/hour for infants ≤72 hours of age or if TSB decreased for infants >72 hours of age. This study was concluded in August 2012, details of which are scheduled for publication shortly. In summary, 203 term and near-term newborns (≤14 days old) with clinically significant jaundice as assessed by TSB levels were enrolled. They received treatment under a FS-PT canopy over a period of 227 days. Hourly measurements of axillary body temperatures and monitoring of sunburn, dehydration, and irradiances of FS-PT were performed. The results showed that FS-PT was efficacious in 94% (164/175) of the infants studied. Average irradiance from FS-PT was 37 ± 14 (range 8 to 65) μW/cm^2^/nm as measured by the Bili-Blanket Meter II^©^ (General Electric, Fairfield, CT, USA). No infant developed sunburn or dehydration, nor met exclusion criteria. Based on these favorable findings with our selected films (Gila Titanium and Air Blue 80 produced by CP Films Inc. Subsidiary of Eastman Chemical Co. Fieldale, VA, USA), we set out under the second phase of this project to demonstrate primarily in a RCT that FS-PT is just as efficacious as CPT in the treatment of infants with or at risk of severe NNJ.

## Methods/design

### Aims and hypotheses

The primary goal of this study is to demonstrate that the efficacy of FS-PT is no worse than that of CPT, with a non-inferiority margin of 10%. In effect, our null hypothesis is that this margin is above 10% suggesting that FS-PT is inferior to CPT.

The secondary goal of the study is to demonstrate that the proportion of infants who require exchange transfusion under FS-PT is no worse than that under CPT, with a non-inferiority margin of 5% based on a clinical rationale that FS-PT is non-inferior to CPT and still better than no treatment. This was also informed by the results of a meta-analysis of RCTs comparing CPT with no treatment which estimated that 10 infants needed to be treated with CPT to prevent an exchange transfusion [26].

### Study design

This is a prospective, non-blinded, single center, randomized two-stage controlled non-inferiority clinical trial. During the first stage of the study, infants will be referred from elevated transcutaneous bilirubin (TcB) screening and enrolled for the definitive TSB estimation. Only infants with significantly elevated TSB levels will qualify for inclusion into the second stage of the study for PT treatment and will be randomly assigned to receive FS-PT or CPT.

### Study setting

The study location is Island Maternity Hospital (IMH), Lagos, Nigeria, which is a state-owned hospital. It has a special care baby unit with CPT and other basic neonatal care facilities, including intravenous (IV) fluids, antibiotics, and exchange transfusion. Ventilator support and pumps for giving IV fluids are not available. It is a 180-bed residency teaching hospital with about 300 deliveries per month of which 45 infants (15%) would be expected to qualify for PT. It is possible at this location to give routine care, IV fluids, antibiotics, oxygen, and medications without interrupting PT. The average maximum temperature in Lagos is 32°C (90°F) during the dry season (November to March), with a maximum recorded temperature of 40°C (104°F), making FS-PT possible even with infants unclothed or minimally clothed. In the rainy season it should be possible to maintain satisfactory irradiances in the absence of an excessively overcast sky. This site was chosen for the maiden study to ensure that back-up neonatal care services are readily available when required until we have the required safety and efficacy data.

### Definition of high-risk threshold

All jaundiced infants with elevated TSB, defined as 3 mg/dl below the level recommended by the American Academy of Pediatrics (AAP) [27] for PT in high-risk infants, will be considered at high risk. The high-risk classification category of the AAP guideline was selected because laboratory evaluation for hemolysis will not be consistently available during the study, and Nigerian infants are known to be at higher risk for severe NNJ secondary to glucose-6-phosphate dehydrogenase (G6PD) deficiency [28]. This TSB level was chosen also because many hospitals begin PT about 2 to 3 mg/dl below the current level in the United States, due to the severity of NNJ and the sub-optimal PT treatment in most locations in Nigeria.

### Primary outcome

The primary outcome for the RCT is efficacy of FS-PT compared with CPT.

#### Evaluation criteria

Change in TSB level per hour of therapy defined as a binary variable. The TSB level will be measured immediately before beginning therapy on a given day and immediately after therapy is completed that day, and the change in bilirubin level in mg/dl per hour of therapy will be calculated. For infants who are less than 72 hours old in the morning, the treatment will be judged efficacious if the bilirubin level rises more slowly than 0.2 mg/dl per hour of therapy that day. For infants who are more than 72 hours old, the treatment will be judged efficacious if the bilirubin level falls. Efficacy will be evaluated only for days in which the treatment was deemed safe (that is, when the infant did not have to be removed from PT due to a need for physician treatment for sunburn or dehydration, persistent temperature instability defined as two or more episodes of temperatures <35.0 or >39.0°C, or failure to return to normothermia (defined as 35.5 to 38.0°C) within 1 hour of being removed from PT). The average efficacy of each type of PT, defined as the proportion of safe days where a given therapy was used in which the therapy was efficacious, will be compared. FS-PT will be considered at least as efficacious as CPT if the average efficacy on days where FS-PT was used is no worse than 10% less than the average efficacy on days where CPT was used.

### Secondary outcome

A secondary outcome for this trial is the proportion of enrolled infants who require exchange transfusion. FS-PT will be considered at least as efficacious as CPT if the proportion of infants who require exchange transfusion under FS-PT is no more than 5% greater than the proportion under CPT.

### Sample size

The minimum sample size was estimated using the following assumptions: 1) the average efficacy of CPT is 80%; 2) the average efficacy of FS-PT is 80%; 3) there will be the same number of infants assigned to both groups; 4) the delta value is 10% - that is, we want to demonstrate that FS-PT is no more than 10% worse than CPT; and 5) 80% power, one-sided alpha = 0.025.

**Figure 2:**
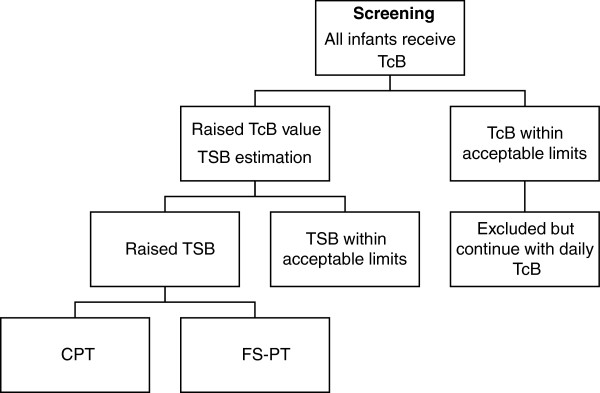
**Enrolment flow chart.** CPT, conventional phototherapy; FS-PT, filtered sunlight phototherapy; TcB, transcutaneous bilirubin; TSB, total serum/plasma bilirubin.

Under these assumptions, a total of 504 days, or 252 days in each treatment group, will be required. This calculation gives the required number of treatment days that are evaluable for efficacy. Since efficacy will be evaluated only for days in which the treatment was deemed safe, the sample size was increased to account for the possibility that the proportion of safe days might be as low as 90%. Assuming that the safety of both FS-PT and CPT is 90%, a sample size of 560 total days is required. Assuming that a typical course of treatment lasts 1 day, both for FS-PT and CPT, then 560 infants, or 280 infants in each group, will be required. Anticipating up to 10% loss due to rainy days (in the FS-PT group), delisting of infants by parents or guardians, or missing data (in both groups), we will enroll 616 infants, or 308 infants in each group. From our year 1 data in the preceding observational study only 66% of the 826 infants referred for elevated TcB had significant elevated TSB levels, hence 915 infants or more will be enrolled for stage 1 bilirubin estimation until the total requirement of 560 treatment days is reached (Figure 2).

### Subject recruitment

Infants born in the hospital will be screened daily from 0 to 14 days of life for elevated TSB using TcB measurements, while in the hospital or, if discharged before 14 days, in the community by nurses sent out from the hospital when possible. Infants from the community will also be screened if within 14 days they choose to come to the study site for screening. The parents will be asked to return with their infants to the hospital if the parent observes new-onset jaundice between 1 and 14 days of life. If the TcB is not elevated during the first 14 days of life, the subject will cease to be eligible for the study. If the TcB is elevated and the other study inclusion and exclusion criteria are met, then the infant will be recruited for the study after informed consent in writing or thumb printing is obtained from a parent or guardian. The infant will then have a TSB measured. Enrolled infants with elevated TSB (defined as 3 mg/dl below the level recommended by the AAP [27] to begin PT in high-risk infants) will be randomly assigned to receive FS-PT or CPT. Enrolled infants with TSB levels that are not elevated as defined above will not be treated with PT. They will continue to be screened for elevated TcB as described above. If their subsequent TcB levels are not elevated during the first 14 days of life, the infant will be withdrawn from the study.

### Subject screening

Screening criteria prior to enrolment are shown in Table 1.

**Table 1:** Screening criteria prior to enrolment

**Test**	**Screening requirement**
Gestational age and/or weight	Greater than or equal to 35 weeks gestation (or >2.2 kg if gestational age is not available). Infant must be ≤14 days old at the time of first PT.
TcB	Screened daily for the first 7 days of life if in hospital and, when possible, in the community if discharged before 7 days of life or if the patient chooses to come in for screening up to14 days of life. If the TcB is elevated (elevated will be defined as a point 3 mg/dl below that recommended by the AAP for PT in high risk infants and above) at any time during the first 14 days of life, a TSB will be done.
Physical examination (dehydration, oxygen use, ABE, life expectancy, sunburn)	Subjects cannot be dehydrated, sunburned, using oxygen, or have ABE or a life expectancy <24 hours at the time of examination by the physician for enrollment in the treatment phase of the protocol.

AAP, American Academy of Pediatrics; ABE, acute bilirubin encephalopathy; PT, phototherapy; TcB, transcutaneous bilirubin; TSB, total serum/plasma bilirubin.

### Prior and concomitant therapy

All infants who meet the AAP guideline for the initiation of PT will be treated with CPT at night or when it is not possible to use FS-PT, secondary to rain or overcast sky. CPT will be performed per hospital standards. Additionally, since the study physicians only serve as consultants for patients requiring treatment for NNJ, patient management decisions, including the need for and timing of exchange transfusion, administration of antibiotics or other testing, will be at the discretion of the attending physician. AAP guidelines will be used to direct the decision of when to perform exchange transfusion, but the final decision will be that of the attending physician. Generally, exchange transfusion is performed in any term infant in Nigeria whose TSB level is ≥20 mg/dl or in any infant with neurologic signs of ABE, regardless of the recommendations in the AAP guideline.

### Inclusion criteria

Subjects will be eligible to participate in the study if all of the following conditions exist:1) at time of birth, infant is ≥35 weeks gestation (or >2.2 kg if gestational age is not available); 2) infant is ≤14 days old at the time of enrollment; 3) at time of enrollment, infant has an elevated TcB defined as 3 mg/dl below the level recommended for high-risk infants per AAP guideline or higher; 4) parent or guardian has given consent for the infant to participate.

### Exclusion criteria

Subjects will be excluded from participation in the study if any of the following conditions exist at the time of enrollment:1) infants with a condition requiring referral for treatment not available at the hospital study site and/or CPT unit; 2) infants with a life-expectancy of <24 hours; 3) infants requiring oxygen therapy; 4) infants clinically dehydrated or sunburned; 5) infants with a temperature <35.5°C or >38°C; 6) infants with ABE on clinical examination; 7) infants meeting the criteria for exchange transfusion.

Inclusion and exclusion criteria are only applicable at the time of enrollment before randomization to prevent bias of the treatment comparison.

### Exit/discontinuation/withdrawal criteria

Subjects will exit the study (will cease to receive FS-PT or CPT) if any of the following conditions occur: 1) persistent temperature instability defined as two or more episodes of temperatures <35.0°C or >39.0°C; 2) failure to return to normothermia (defined as 35.5°C to 38.0°C) within 1 hour of being removed from PT; 3) physician treatment for dehydration or sunburn is required; 4) TSB level reaches exchange transfusion levels; 5) inter-current illness not compatible with PT or needing more care than can be provided in the FS-PT canopy or CPT ward; 6) infants requiring transfer to another hospital; 7) parent request; 8) subject death; 9) subject completes the protocol (TcB or TSB level is no longer elevated); 10) subject’s well-being, in the opinion of the Investigator, would be compromised by study continuation; 11) Institutional Review Board (IRB) recommendation; or 12) subject’s risk level indicates PT should be stopped.

Infants who have been withdrawn from the study will have a physical examination and a final TcB. Safety data will be analyzed for all days in which the subjects spend any time under either FS-PT or CPT.

Efficacy will be evaluated for all days in which the treatment was deemed safe (that is, when the infant did not have to be removed from PT due to a need for physician treatment for sunburn or dehydration, due to persistent temperature instability defined as two or more episodes of temperatures <35.0 or >39.0°C, or due to failure to return to normothermia (defined as 35.5 to 38.0°C) within 1 hour of being removed from PT).

### Randomization scheme

Enrolled infants with elevated TSB (defined as 3 mg/dl below the level recommended by the AAP to begin PT in high-risk infants) will be randomly assigned to receive FS-PT or CPT. A block randomization procedure with variable block sizes will be used to maximize unpredictability. The randomization assignments of FS-PT or CPT will be printed on sequentially numbered sheets of paper and enclosed in opaque, sealed, sequentially numbered envelopes. The sealed envelopes will be prepared independently by the study statistician based in the USA and transported to Nigeria by the regulatory sponsor. When an enrolled infant’s TSB level is obtained and found to be 3 mg/dl or more below the AAP threshold for high-risk infants the study nurse will request the next envelope. The envelope will be opened and both the envelope number and the treatment assignment will be recorded on the case report form. The laboratory technician responsible for measuring the serum bilirubin level will be unaware of the sequence of treatment allocation of either FS-PT or CPT for each eligible infant and will not be involved in administering PT. The study will not be blinded as it is not physically possible to blind either the participating infants or parents, nor the hospital personnel.

### Laboratory testing procedures

#### *G*lucose-6-phosphate dehydrogenase

Because of the high prevalence of G6PD deficiency in our study population, enrolled infants will be routinely screened for this condition using simple and inexpensive supplies. The method will be based on the fluorescent blood spot originally developed by Beutler and colleagues [29]. This test has been considered a standard method of G6PD screening especially in resource-poor settings, since inexpensive reagents and materials can be used.

#### Bilirubin

TSB will be estimated using standard methods. Hematocrit testing will be done using standard laboratory methods.

#### Coombs testing and blood typing

Coombs testing and blood typing will be performed in the blood bank laboratory at IMH.

#### Specimen collection

Blood specimens for TSB measurements will generally be collected by heel-stick. Specimens for G6PD, blood type and Rh will be drawn by venipuncture.

### Clinical procedures

#### Criteria for admission

Infants born in the hospital will be screened daily from 0 to 14 days of life for elevated TcB while in the hospital or, if discharged before 14 days, in the community by nurses sent out from the hospital when possible. Infants ≤14 days old from the community presenting to the study site will also be screened. The parents will be asked to return with their infant to the hospital if the parent observes new onset jaundice between 1 and 14 days of life.

#### Screening

During the screening period, information about the baby and the mother will be collected. TcB testing will be done for up to 14 days, using Minolta AirShields Jaundice Meter JM-103 (Draeger Medical Systems, Inc., Telford, PA, USA) either in the hospital or in the community. If the TcB is not elevated during the first 14 days of life, the subject will not be eligible for the study. If at any time during screening the infant’s TcB level rises to within 3 mg/dl below the AAP threshold [27] for high-risk infants, inclusion/exclusion criteria will be reviewed. If the infant is eligible, the infant will be recruited for the study. The infant’s mother and/or father will be approached to ask if they are interested in participating in the research study. If they are willing, the consent form for the study will be discussed. If informed consent is given to participate, consent will be documented on the consent form in writing or thumb printing.

#### Pre-treatment (stage 1)

The enrolled infant will then have a TSB drawn (stage 1) using the Advanced Bilirubinometer Stat-Analyzer, Model BR2 (Advanced Instruments, Inc, Norwood, MA, USA) before treatment (stage 2). Enrolled infants with elevated TSB qualify for inclusion into the second stage of the study and will be randomly assigned to receive FS-PT or CPT. Enrolled infants with TSB levels that are not elevated as defined above will not proceed to the second stage to be treated with PT. However, they will continue to be screened for elevated TcB as described above. If their subsequent TcB levels are not elevated during the first 14 days of life, the infant will be withdrawn from the study. All infants who meet the AAP guideline for the initiation of PT will be treated with CPT at night or when it is not possible to use FS-PT, secondary to rain or excessive cloud cover (defined as cloud cover persisting for more than 2 hours). FS-PT will be optimized in all infants by using white cloth lining the bottom and sides of the cot and exposing the infant maximally. CPT will be performed per international standards of practice [27].

#### Treatment (stage 2)

FS-PT will be started in the morning after the irradiance level inside the canopy is at least 8 μW/cm^2^/nm, and will be stopped in the late afternoon when the irradiance level drops below 8 μW/cm^2^/nm or at any point during the day during a rainy/cloudy day when the irradiance stays below 8 μW/cm^2^/nm. If the irradiance persists below 8 μW/cm^2^/nm for more than 1 hour and the infant qualifies for PT per the AAP guideline, CPT will be initiated. If the irradiance again exceeds 8 μW/cm^2^/nm then the infant may again be placed in FS-PT.

#### Treatment canopies

Two FS-PT canopies will be used: one fitted with the Gila Titanium film and the other fitted with the Air Blue 80 film because of concerns about irradiance and heat. The Titanium canopy transmits approximately 33% blue light in the wavelength range 400 to 520 nm with much lower heat and will be used during sunny periods of the day. The Air Blue canopy transmits roughly 79% blue light as well as heat and will be used during cooler overcast periods of the day. The infants will be moved from one canopy to the other as needed during the day, when the weather changes, with the goal of keeping the irradiance level above 8 μW/cm^2^/nm. The irradiance will be measured at an infant’s abdomen level with a Bili-Blanket Meter II every half hour in infants under FS-PT (if possible, additional measurements may be done) and daily in those infants under CPT.

The proportion of blue light accessible to the subjects under FS-PT canopies is dependent on the location of treatment canopies in relation to surrounding buildings. It is therefore imperative that the treatment canopies be placed in a location with proven day-long sun exposure throughout the year, preferably one also protected from potentially violent wind currents and surface dust generated by traffic. In fact, the single most important reason for replacing films is wind damage. A roof top terrace, presently in use in our study, has proven to be a very satisfactory location. A courtyard of adequate size surrounded by low buildings could be even more suitable. Finally, perhaps most ideal and practical, will be the construction of a suitable FS-PT room or a small permanent building with a glass or plexiglass roof, in an appropriately “sunny” part of a hospital. This would be a most ideal solution to prevent issues with wind, dust, security, and so forth. However, such a treatment facility may overtax local resources.

#### Treatment duration

Generally, the infants will be placed under FS-PT/CPT from 9:00 am to 5:00 pm (target minimum duration of 5 hours per day for any infant in the study at 10:00 am, or 65% of total time between start time and 5:00 pm for infants enrolled after 10:00 am). The infants’ eyes will be protected with low-cost eye covers made from the elastic tops of any color socks. Bilirubin kinetics will only be calculated for infants who are able to spend at least 5 hours in FS-PT/CPT.

#### Temperature measurements

Infant temperature will be measured hourly while under FS-PT and CPT and monitored based on World Health Organization guidelines for normothermia, hypothermia, and hyperthermia [30]. For the purpose of this study the normal temperature range of 36.5 to 37.5°C [30] was modified slightly to 35.5 to 38.0°C in consultation with a neonatologist to provide a broader but safe temperature limit for term and near-term infants. In addition, during the prior safety and efficacy observational study in which infants were monitored closely, no untoward effects of temperatures within the chosen range were reported. If the axillary body temperature falls below 35.5°C the infant will be placed skin-to-skin with the mother and/or brought inside until the temperature returns to the normal range of 35.5 to 38.0°C. If the axillary body temperature goes above 38.0°C, the infant will be brought indoors and/or placed in the shade and/or placed on a wet towel until the body temperature returns to normal at which time FS-PT will resume. The time it takes to return to normal will be recorded. Infants who do not return to normothermia within 1 hour will be taken out of FS-PT for that day. If the axillary body temperature falls below 35°C the infant will be placed skin-to-skin with the mother or wrapped well and brought inside until the temperature returns to normal. If the axillary body temperature goes above 39.0°C, the infant will be brought indoors and placed on a wet towel until the body temperature returns to normal at which time FS-PT will resume. The time it takes to return to normal will be recorded. Infants who do not return to normothermia within 1 hour will be taken out of FS-PT for that day. Infants whose axillary body temperature fall below 35°C or exceed 39°C on two occasions will be excluded from the study.

#### Dehydration

The nurse will also check hourly for clinical signs of dehydration in the form of dry mucus membranes, sunken eyes, dry eyes, and skin tenting. The physician may elect to increase the mother’s breastfeeding frequency while continuing with the FS-PT if the diagnosis is mild dehydration. Whenever the diagnosis of moderate or severe dehydration is made, the infant will be withdrawn from the study for treatment. Because both nutrition and hydration are important in the prevention and treatment of severe NNJ, infants will be allowed and encouraged to breastfeed *ad lib* and often. The FS-PT canopy allows for the mother to stay with the infant and to feed as often as she desires. Mothers will be comfortably seated on custom-made reclined chairs (painted white) and provided with white aprons for breast-feeding or when they need to carry their babies (Figure 1C). If the mother has chosen to use artificial feeds, those will also be allowed and encouraged *ad lib*. For CPT, the mother will be allowed and encouraged to take the infant out as needed for bathing or feeding.

#### Sunburn

The nurse will assess for sunburn by looking for the onset of pink skin every hour. Most infants are born relatively light skinned and darken over time. Infants born very dark and for whom it would be difficult to recognize pink skin are also much less likely to become sunburned. Absence of sunburn determined in consultation with a dermatologist is defined as the absence of new-onset pink skin. If the infant has the onset of pink skin, the attending physician will be notified and will verify the finding. If the physician makes the diagnosis of sunburn, the infant will be immediately withdrawn from the study. Mothers will be allowed and encouraged as much as possible to stay with their babies, but will be able to leave as needed to care for themselves or other needs. Study nurses will care for the infants during their mother’s absence.

#### Treatment efficacy

Efficacy will be tested by measuring TSB levels prior to the placement of the infant under PT each morning to establish baseline TSB levels. Infants with morning TSB levels that meet the criteria for PT will be randomized for FS-PT. Repeat TSB levels will be obtained at the end of each day spent under FS-PT and between ~4:00 to 5:00 pm for infants under CPT to estimate the effectiveness of the treatment for the day and to determine if the requirement for night-time PT is met.

### Data collection and analysis

Data will be collected on paper case report forms and entered via a web interface into a secure database. The study data will be collected and managed using REDCap (Research Electronic Data Capture) electronic data capture tools hosted at the University of Minnesota’s Academic Health Center. REDCap is a secure, web-based application designed to support data capture for research studies, providing: 1) an intuitive interface for validated data entry; 2) audit trails for tracking data manipulation and export procedures; 3) automated export procedures for seamless data downloads to common statistical packages; and 4) procedures for importing data from external sources.

Demographic data will be summarized and compared between the treatment and control groups using appropriate methods, such as Pearson’s chi-square test for categorical variables and two-sample t-tests for continuous variables. The primary objective is to compare the efficacy of FS-PT and CPT. A 95% exact binomial confidence interval for the true difference in efficacy between the FS-PT treated and CPT control groups will be calculated. FS-PT will be deemed non-inferior to CPT if the calculated confidence interval does not extend more than 10% below the equivalence point.

We plan to conduct three distinct analyses: an intent-to-treat analysis based on the assigned treatment for each enrolled infant, an as-treated analysis based on the actual treatment received on a given day and a per-protocol analysis including only enrolled infants who received the assigned treatment on all of their treatment days.

### Safety monitoring and adverse events

The Principal Investigator will oversee the safety of the study, including careful assessment and appropriate reporting of adverse events to relevant authorities at IMH. All anticipated adverse events such as sunburn, dehydration, and temperature instability as well as unanticipated adverse events occurring during the study period will be recorded and closely monitored. Medical monitoring will include a regular assessment of the number and type of serious adverse events. The clinical course of each event will be followed until resolution, stabilization, or until it has been determined that study treatment or participation is not the cause.

All observed or volunteered adverse effects and abnormal test findings, regardless of treatment group, if applicable, or suspected causal relationship to the investigational device or, if applicable, other study treatment or diagnostic product(s) will be recorded in the subjects’ case histories. For all adverse effects, sufficient information will be pursued and/or obtained so as to permit: 1) an adequate determination of the outcome of the effect (that is, whether the effect should be classified as a serious adverse effect); and 2) an assessment of the casual relationship between the adverse effect and the investigational device or, if applicable, the other study treatment or diagnostic product(s).

### Ethical considerations

This study was approved by the IRBs/Ethical Committees at the University of Minnesota, Minnesota Medical Research Foundation (Hennepin County Medical Center), and the Lagos State Government. This study is being conducted according to United States and international standards of Good Clinical Practice (21 CFR 812 and International Conference on Harmonization guidelines), applicable government regulations and Institutional research policies and procedures. The parents/guardians of potential subjects for this study will be provided a consent form describing this study and providing sufficient information for the parents/guardians to make an informed decision about their child’s participation in this study. The consent form has been submitted with the protocol for review and approval by the relevant IRBs/Ethical Committees for the study. The formal consent of a parent/guardian, using the IRB-approved consent form, will be obtained before the infant is subjected to any study procedure. This consent form will be signed or thumb-printed by the parent/guardian, and the investigator-designated research professional obtaining the consent. A blank copy of the IRB-approved form will be kept on-site and by the sponsor-investigator.

## Trial status

The first participant was randomized on 29 November 2012 and recruitment is ongoing as of 31 July 2013.

## Abbreviations

AAP: American Academy of Pediatrics; ABE: Acute bilirubin encephalopathy; CPT: Conventional phototherapy; FS-PT: Filtered sunlight phototherapy; G6PD: Glucose-6-phosphate dehydrogenase; IMH: Island Maternity Hospital; IRB: Institutional Review Board; IV: Intravenous; NNJ: Neonatal jaundice; PT: Phototherapy; RCT: Randomized controlled trial; REDCap: Research Electronic Data Capture; TcB: Transcutaneous bilirubin; TSB: Total serum/plasma bilirubin.

## Competing interests

The authors declare that they have no competing interests.

## Authors’ contributions

TMS, BOO, HJV, RJW, AMB, YEV and DKS contributed substantially to the conception, design and implementation of this trial. BOO and TMS drafted the manuscript. TMS, BOO, HJV, RJW, AMB, YEV and DKS reviewed the manuscript critically for intellectual content. All authors read and approved the final version before submission.
